# Supplementary figures and images for: Functional differentiation of human dental pulp stem cells into neuron-like cells exhibiting electrophysiological activity
Source: Stem Cell Res Ther. 2025 Jan 23;16:10. doi: 10.1186/s13287-025-04134-7 (PMC11756023; doi:10.1186/s13287-025-04134-7)

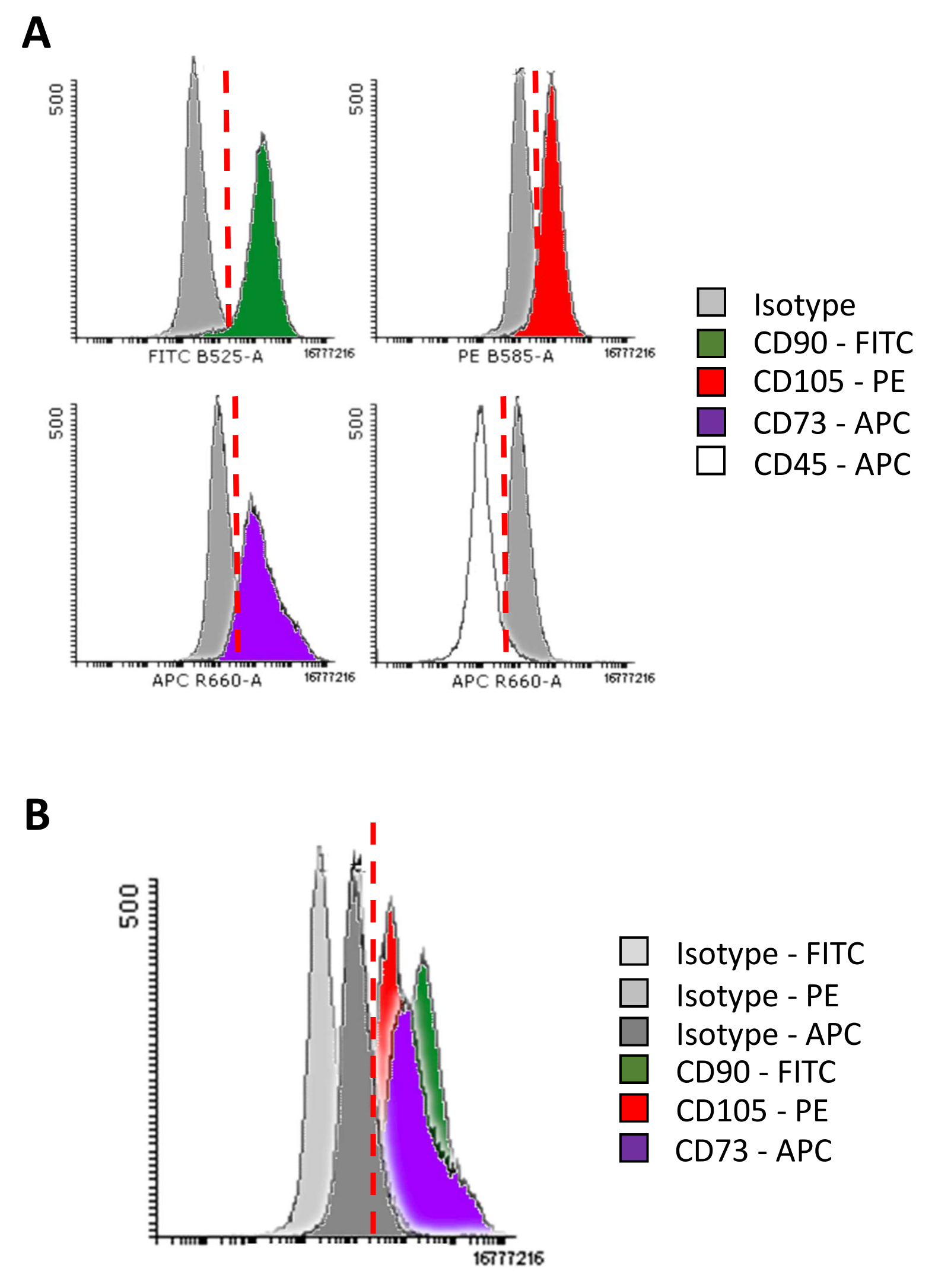

Supplement: Supplementary file 4 — Supplementary Material 4: Nuclear area measured after 21 days of neural induction process. Graph showing a larger nuclear area (µm2) in cells cultured with FBS after measuring 100 nuclei per experimental condition in 3 different donor’s cell cultures with the NII plugin for Image J. Data shown as mean ± SD. ***p < 0.001. Mann-Whitney U test (Two-tailed). [file 13287_2025_4134_MOESM4_ESM.tif]

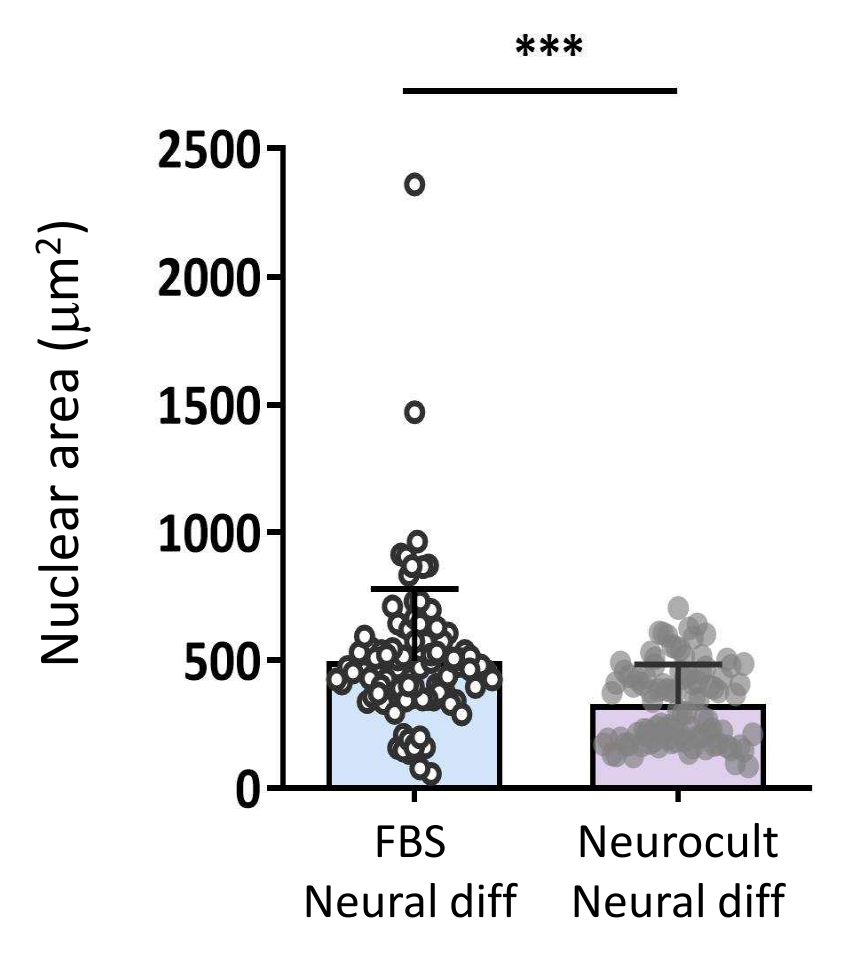

Supplement: Supplementary file 5 — Supplementary Material 5: Expression of Mesenchymal Stem Cell Markers. (A-B) Flow cytometry analysis for mesenchymal stem cell markers (CD90, CD105, CD73, CD45) confirmed a generalized ectomesenchymal phenotype in hDPSCs cultured in DMEM with 10% FBS as an adherent monolayer. [file 13287_2025_4134_MOESM5_ESM.tif]

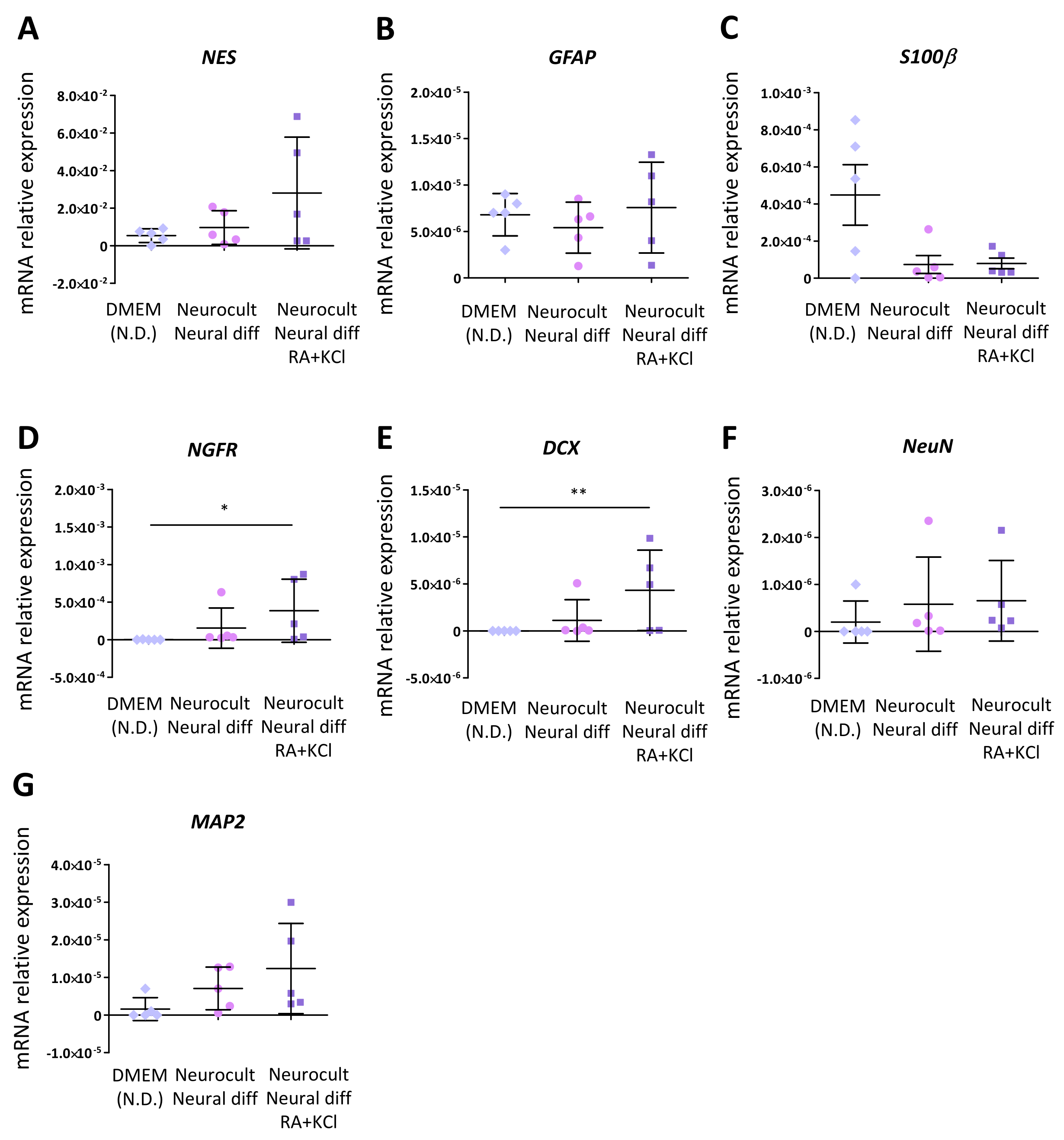

Supplement: Supplementary file 6 — Supplementary Material 6: mRNA expression levels in non-differentiated (N.D.) hDPSCs and after RA and KCl addition to Neurocult differentiation mix at 21 days of neurodifferentiation. RT‒qPCR analysis of (A) Nestin, (B) GFAP, (C) S100β, (D) NGFR and neuronal (E) DCX, (F) NeuN and (G) MAP2 relative mRNA expression in hDPSCs cultures from 5 different donors treated or not with RA plus KCl. Data shown as mean ± SD. * p < 0.05, ** p < 0.01. Statistical analysis performed by Kruskal-Wallis test followed by Dunn’s multiple comparisons test. [file 13287_2025_4134_MOESM6_ESM.tif]

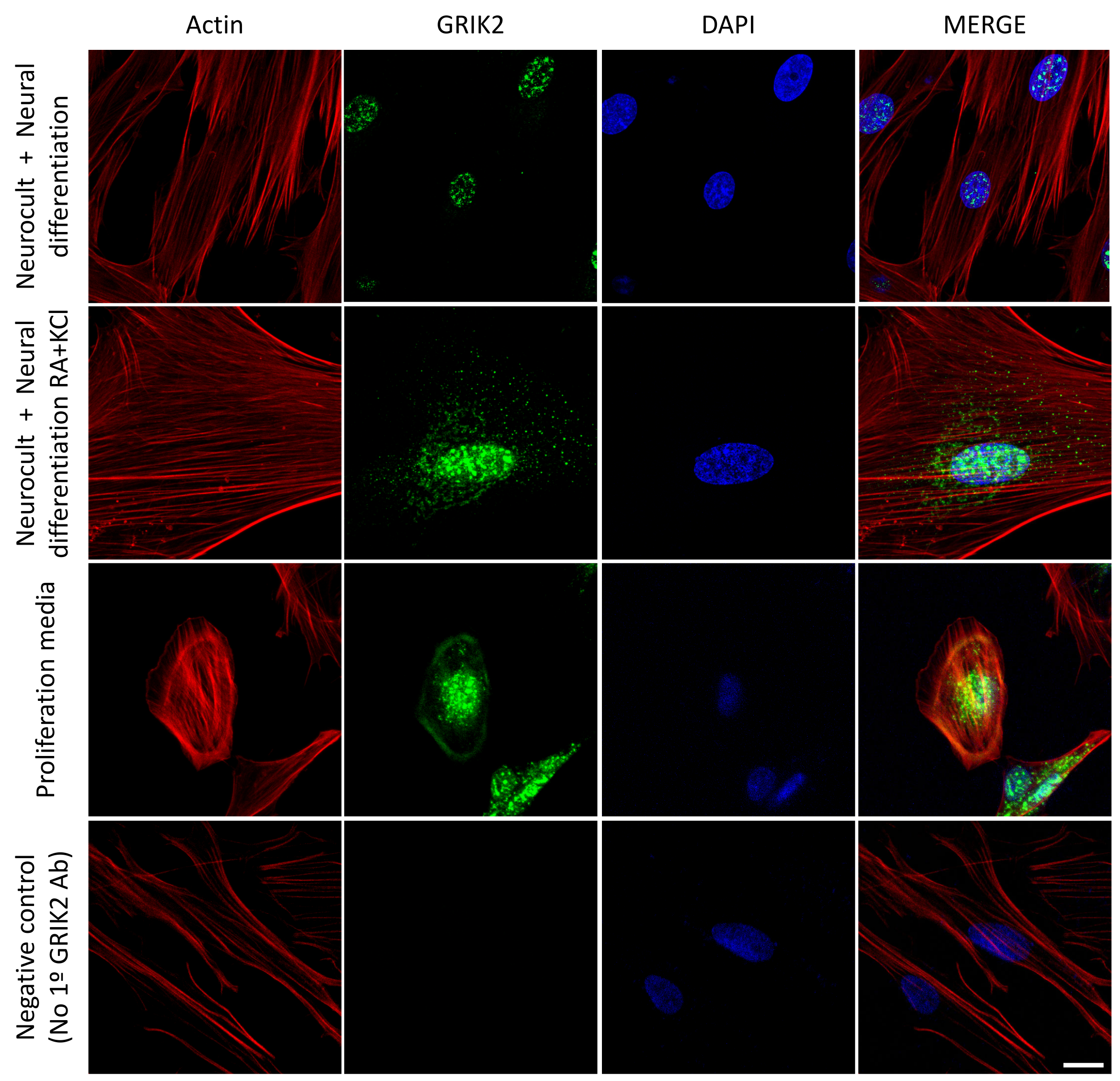

Supplement: Supplementary file 7 — Supplementary Material 7: GRIK2 immunostaining in differentiated hDPSCs after 21 days with or without RA + KCl or hDPSCs in proliferating media. Confocal Immunofluorescence images showing the cell membrane and cytoplasmatic region using Phalloidin staining (red) and GRIK2 protein expression (green) showing a membrane, intracytoplasmic and nuclear staining. Negative control staining without GRIK2 primary antibody. Scale bar 20 μm. [file 13287_2025_4134_MOESM7_ESM.tif]

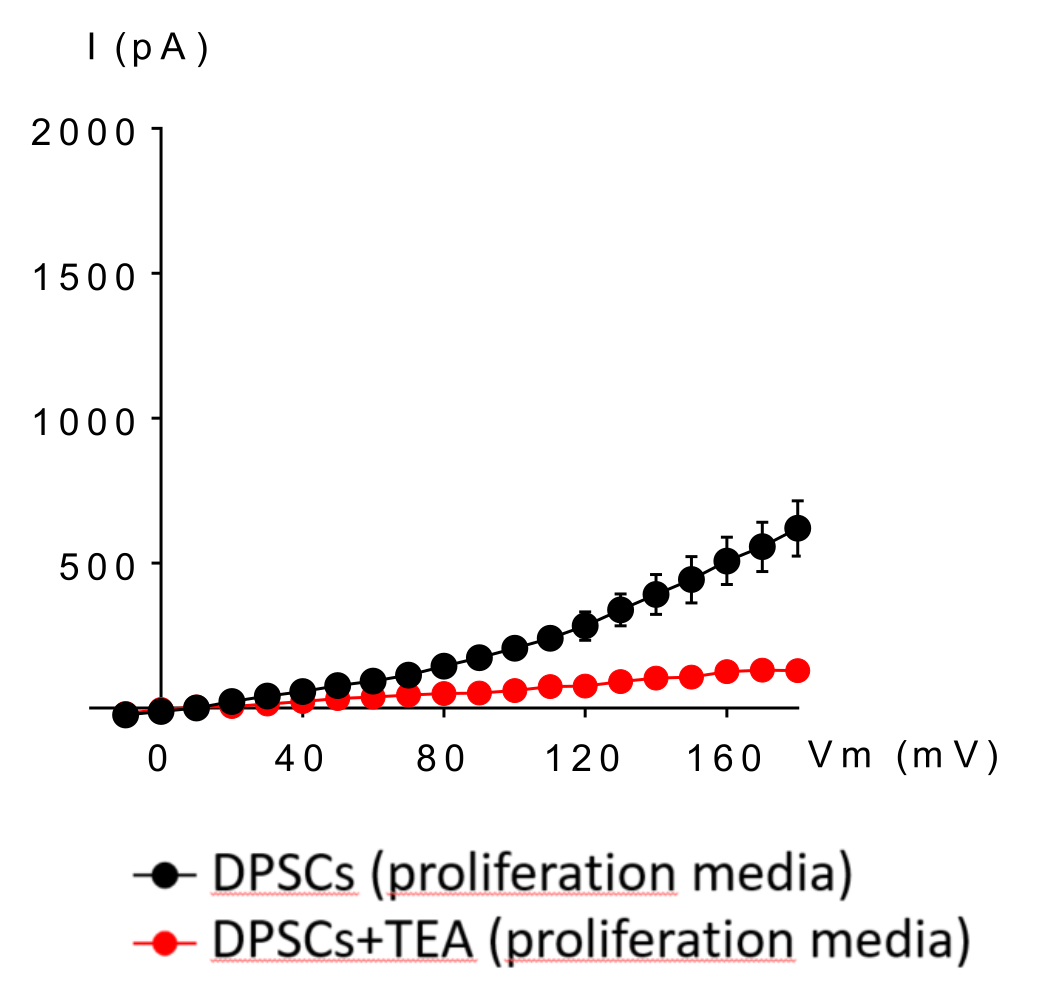

Supplement: Supplementary file 8 — Supplementary Material 8: Electrophysiological recording of voltage-dependent K+ currents of non-differentiated hDPSCs. Non-neurodifferentiated hDPSC grown in proliferation media showed voltage-dependent K+ currents, which could be largely blocked using TEA. [file 13287_2025_4134_MOESM8_ESM.tif]
